# Development of a novel protocol for processing fluorescent microspheres used in quantifying tissue perfusion

**DOI:** 10.1016/j.sbsr.2025.100865

**Published:** 2025-08-27

**Authors:** Michael B. Nelappana, Catherine C. Applegate, Leopold J.B. Pinot, Elaine A. Nielsen, Karl Baumgartel, Goodluck Okoro, Leszek Kalinowski, Iwona T. Dobrucki, Lawrence W. Dobrucki

**Affiliations:** a Department of Bioengineering, University of Illinois at Urbana–Champaign, Urbana, IL 61801, USA; b Beckman Institute for Advanced Science and Technology, University of Illinois at Urbana–Champaign, Urbana, IL 61801, USA; c Cancer Center at Illinois, University of Illinois at Urbana-Champaign, Urbana, IL 61801, USA; d Division of Medical Laboratory Diagnostics - Fahrenheit Biobank BBMRI.pl, Medical University of Gdansk, Gdansk, Poland; e BioTechMed Centre, Department of Mechanics of Materials and Structures, Gdansk University of Technology, Gdansk, Poland; f Department of Biomedical and Translational Sciences, Carle-Illinois College of Medicine, University of Illinois at Urbana-Champaign, Urbana, IL 61853, USA; g Academy of Medical and Social Applied Sciences, Elblag, Poland

**Keywords:** Microspheres, Peripheral arterial disease, Fluorescent imaging, Tissue perfusion, Fluorometry

## Abstract

Alteration of blood perfusion leads to some of the most common cardiovascular pathologies. Current methods for measuring perfusion use fluorescent polystyrene microspheres (MS) that are systemically injected prior to processing to obtain the absolute number of MS trapped inside the tissue. The current standard method is cost-intensive and carries a high risk of MS loss, leading to underestimation of regional perfusion. This study aimed to develop an improved, cost-efficient protocol for measuring regional perfusion through the processing and direct imaging of fluorescent MS embedded ex vivo. Porcine and control samples treated with MS were chemically digested, filtered through either a polycarbonate (PCTE) or cellulose filter, and fluorescence was measured either through the standard fluorometric method or through the proposed direct imaging method. In the standard fluorometric method, interactions were found between the PCTE filter and porcine samples, leading to dampened signal and the subsequent underestimation of regional perfusion in practice. The proposed direct imaging method with cellulose filters showed improved sensitivity even within low MS levels (limit of detection improved significantly), amplification of sample fluorescence (11–13× when compared to PCTE filters), parity between porcine and control samples, and a reduction in cost providing a significant improvement over the industry standard for fluorescent MS perfusion measurement (28–51 % reduction compared to standard method). The proposed method also removed the need for 2-ethoxy ethyl acetate, a teratogen and plastic softener, and reduced complexity in the workflow.

## 1. Introduction

Blood perfusion is a fundamental biological process essential for the proper functioning of tissues and organs, providing nutrients and oxygen via systems of vasculature, including capillaries and other blood vessels. Alteration of this essential process leads to some of the most common cardiovascular pathologies, such as coronary arterial disease and peripheral artery disease [1,2]. Due to the prevalence of these diseases, further research into diagnostic tests and treatments for inadequate perfusion is needed, including the development of methods to assess tissue perfusion.

Clinically, there are three major types of tissue perfusion measurement: perfusion CT, perfusion MRI, and nuclear imaging. Dynamic perfusion CT detects the passage in vessels and uptake in tissues of an iodinated contrast agent over time, which can then be studied to develop 3D quantitative perfusion maps of a region of interest, such as an organ [3]. Perfusion MRI consists of three techniques: contrast-enhanced MRI, which is the only clinically available method and, like perfusion CT, detects contrast agent passage through tissues [4]; arterial spin labeling, which tags protons in arterial blood through radiofrequency pulses and determines perfusion as the difference between a control MRI and the ‘tagged’ MRI [5]; and blood oxygen level-dependent MRI which measures the local content of deoxygenated hemoglobin which acts as a paramagnetic MRI contrast agent [6]. Nuclear imaging provides a high-sensitivity approach to perfusion detection, typically using either ^15^O-water or a targeted ^99m^Tc radiotracer to be imaged with PET or SPECT, respectively [7,8]. Each of these techniques, however, do not output perfusion values so they must be calibrated against an established perfusion measurement method.

The standard method used in in vivo models involves measuring perfusion of organs and tissues with labeled microspheres (MS) [9]. This technique relies on a simple concept: injected MS, which are typically of diameter of several micrometers, are too large to return to circulation via the lymphatic system, so MS distribution within different tissues is proportional to the perfusion of those regions. This can then be quantified through the detection of the color, fluorescence, or radioactive label within the tissue sample and indirectly counting the number of present MS. Whilst the earliest studies utilized radioactive MS, concerns over the use of ionizing radiation and experimental challenges led to the increased popularity of fluorescent MS which are significantly cheaper and provide better retention in tissue but are more tedious to process compared to radiolabeled MS [10–13].

Current protocols detail the use of 10–15 μm diameter polystyrene beads that are injected directly into the heart’s atrium or left ventricle. After enough time for MS circulation and embedding into the tissue, the animal is euthanized, and the tissues of interest are harvested and processed to obtain the absolute number of MS trapped inside the tissue [14]. Negative pressure filtration traps the MS, which are then chemically burst to release the fluorescent dye. Chemically bursting the MS requires the use of 2-ethoxy ethyl acetate, a solvent that corrodes polystyrene, a commonly used plastic in laboratory equipment, which in turn needs specialized equipment to handle. After the fluorescent dye is released, the fluorescent signal is measured, and the perfusion is related to the strength of the signal. Within the field it is generally accepted that studies require a minimum of 400 MS per tissue sample to accurately measure regional perfusion [15]. This standard method of processing filtered samples of fluorescent MS-embedded tissues is expensive, requires specialized equipment, and carries a high risk of MS loss due to the multiple transfers of the filters and MS to allow for bursting and fluorescent reading which can lead to underestimation of regional perfusion.

**Fig. 1.**
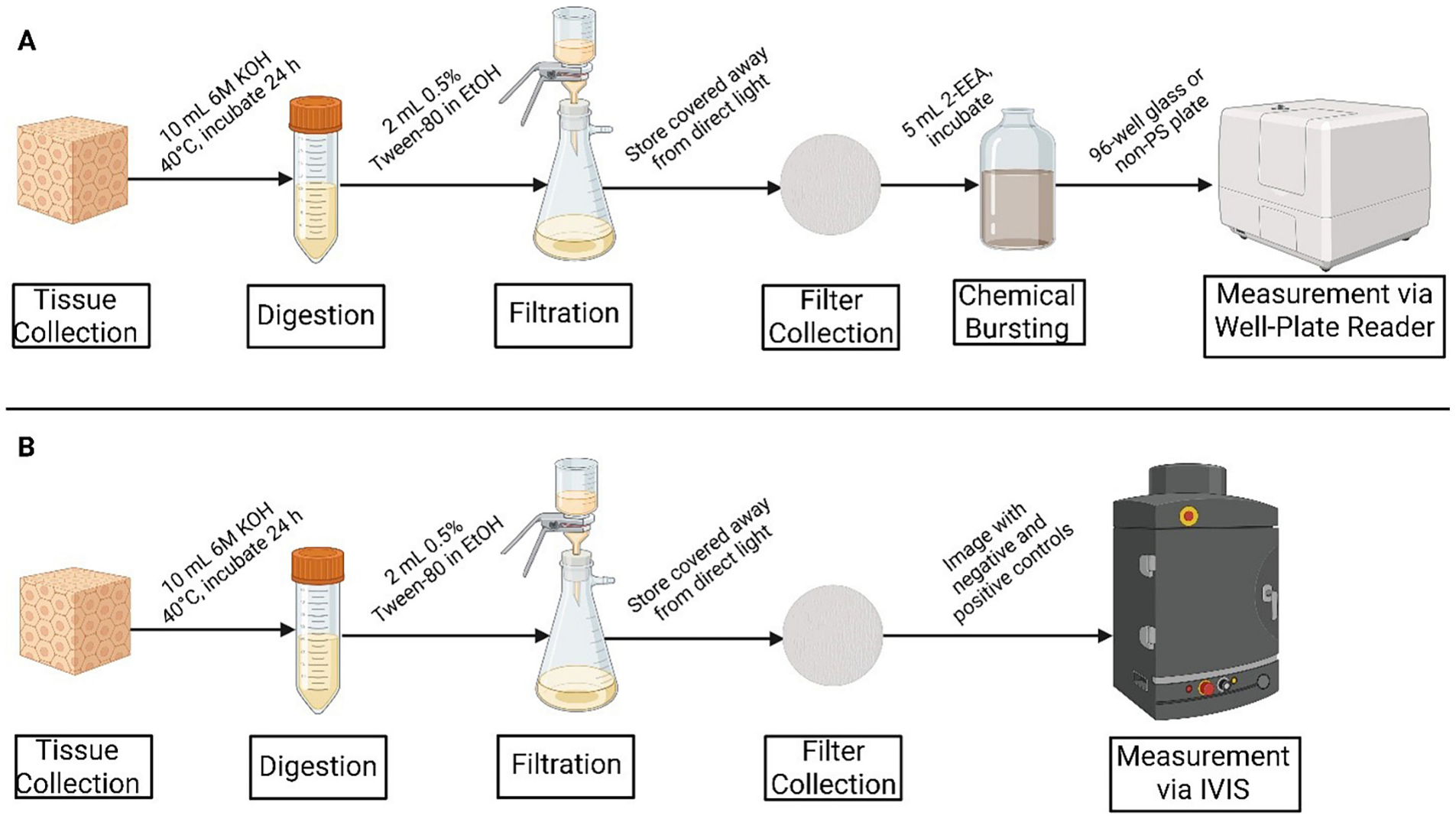
Standard and proposed workflows. Workflow representation of the a) conventional standard fluorometric and b) proposed direct imaging methods. Ethanol = EtOH; 2-ethoxy ethyl acetate = 2-EEA; In Vivo Imaging System = IVIS; polystyrene = PS; potassium hydroxide = KOH. Created with BioRender.com.

The major goal of this study is to develop and optimize an improved protocol for measuring regional perfusion through the processing of fluorescent MS embedded ex vivo. By eliminating the MS bursting process and directly using fluorescence imaging to quantify the intact MS trapped within the filters in this described protocol, we expect to gain a significant reduction in cost; minimization of possible MS loss through the elimination of unnecessary steps (Fig. 1); and similar or better detection sensitivity as compared to the standard method. In addition, we compare two different types of filters in this study: (1) the conventional and more expensive plastic (polycarbonate) filter and (2) an inexpensive, readily available cellulose filter. We expect to see minimal difference in sensitivity between the materials, further reducing the cost of analyzing MS uptake in vascular imaging through the use of our novel protocol.

## 2. Materials and methods

### 2.1. Materials

#### 2.1.1. Reagents/filters

- Fluorescent Persimmon – *Dye-Trak ‘F’* microspheres (Triton Technology, cat. no. 140–575)
- 5.0 μm Hydrophilic Polycarbonate Track-Etched Membrane Disc Filter, 25 mm (GVS North America, cat. no. 1215631)
- 6 μm Grade 3 Qualitative Filter Paper Standard Grade, circle, 23 mm (Cytiva, cat. no. 1003–323)
- Ethyl alcohol, ethanol, pure (Aldrich, cat. no. 459844)
- Phosphate-Buffered Saline, PBS, 1× without calcium and magnesium, pH 7.4 (Corning, cat. no. 21–040-CV)
- Tween-80 (Aldrich, cat. no. P4780)
- Porcine muscle tissue (Local commercial vendor)
- 2-ethoxy ethyl acetate (Aldrich, cat. no. 109967)
- Hydrochloric acid, HCl, 37 % (Fisher Chemical, A144–500)
- 96 Well Round Bottom Plate, PP, 500 μL well (Fisher, cat. no. 12565502)

#### 2.1.2. Solutions

- Digestion Solution: Potassium Hydroxide, KOH, 6 M solution in water, 35–40 °C (Aldrich, cat. no. 221473)
- Digestion Aid Buffer: Tween-80, 0.5 % *w*/*v* solution in ethanol
- Wash Buffer: Tween-80, 0.5 % w/v solution in PBS
- Storage Solution: Hydrochloric acid, HCl, 1:9 (3.7 %) solution in water

#### 2.1.3. Hardware/software

- Quanta FEG 450 Environmental Scanning Electron Microscope (ESEM) (ThermoFisher)
- Millipore Glass Microanalysis Filter Holder Kit (Aldrich, cat. no. XX1012500)
- BioTek Synergy H1 Multimode Microplate Reader (Agilent Technologies)
- IVIS Spectrum In Vivo Imaging System (PerkinElmer cat. no. 124262)
- ImageJ
- SAS (OnDemand for Academics)
- GraphPad Prism (GraphPad Software)

### 2.2. Characterizing microspheres and filters

For all studies described herein, a vial of fluorescent persimmon MS (Excitation/Emission: 540/560 nm; 10–15 μm diameter) with a concentration of 10^6^/mL was utilized. To confirm shape and intactness, the MS were placed on a glass slide and imaged via light microscopy.

Polycarbonate track-etched (PCTE) membrane disc filters and Whatman Grade 3 qualitative filters (CELL) were used to filter fluorescent persimmon MS. These filters were then imaged using an ESEM in the low-vacuum setting to determine and compare surface characteristics and to reveal MS filtering interactions with both filter types. The diameter of the MS was also confirmed using ImageJ with ESEM images of PCTE-filtered MS.

### 2.3. Preparation of microsphere concentration gradient

A stock vial containing 10 million fluorescent persimmon MS was vortexed to disperse any sedimented and clustered MS. To prepare serial dilutions of MS and to emulate the range of MS potentially found in a tissue sample, the stock solution was distributed into DI water to form a concentration of 5000 MS in 100 μL and then serially diluted by a factor of 2 to form a concentration gradient. To improve resolution, a concentration of 4000 MS in 100 μL was also formed and serially diluted by a factor of 2.

### 2.4. Preparation of tissue-microsphere samples

Porcine muscle tissue (POR) was sectioned into ~1 g samples and placed inside of 50 mL centrifuge tubes. Ten mL of freshly made 6 M KOH was added to each tube, still warm (~35–40 °C) from preparation, to facilitate tissue digestion. The centrifuge tubes were placed in racks on a shaking platform in a dark space for 24 h to fully dissolve tissues. Two mL of ethanolic Tween-80 was then added to each sample to dissolve undigested fat, and the solution was vortexed vigorously until clear, and the gradient of MS was added individually to the samples. Control (CON) samples were prepared identically to POR samples, without using porcine tissue.

### 2.5. Filtration of tissue-microsphere samples

The Millipore Glass Microanalysis Filter Holder Kit was assembled to perform negative pressure filtration. A PCTE or CELL filter was placed on the glass frit base, the graduated cylinder was clamped to the base, and the apparatus was connected to suction. Each MS-containing sample was vortexed and poured into the filtration apparatus carefully to ensure that the resulting filtrate wasn’t aspirated into the vacuum. The sample tube and the sides of the graduated cylinder were washed using two 5 mL wash buffer rinses to ensure maximum MS capture. After all the solutions and rinses were filtered, suction and the graduated cylinder were removed, and the filter was transferred to a weigh boat. During the transfer, care was taken to touch only the edges of the filter that contained no MS with forceps, and the labeled weigh boat was covered to block it from light exposure [16]. After all samples were filtered and the full apparatus was washed, the glass frit base was stored in 10 % aqueous hydrochloric acid to prevent protein buildup [17]. Filters were stored in a cool, dark place until they were quantified using either the standard fluorometric or direct imaging method detailed below.

### 2.6. Standard fluorometric measurement of embedded microsphere samples

To complete the standard protocol for fluorometric measurement of MS-embedded samples, MS are required first to undergo a bursting procedure to release fluorescent dyes. Filters with 0, 312, or 5000 MS embedded on the surface were transferred to labeled glass vials with non-polystyrene caps. To each of these vials, 5 mL of 2-ethoxy ethyl acetate was added via glass pipette to dissolve the polystyrene coating making up the exterior layer of MS. Glass vials were placed into a dark box and incubated for 7 days to ensure that all MS ruptured and released fluorescent dyes. From each sample, 150 μL was collected and placed into a non-polystyrene 96-well spectroscopic plate. The well plate was then placed into BioTek Synergy H1 Multimode Microplate Reader, and each sample’s fluorescent intensity (INT) was measured.

### 2.7. Direct imaging of embedded microsphere samples

Each filter was placed inside the IVIS alongside additional control filters that were used to filter an MS-only or a digested POR-only solution. The sample was imaged and subsequently processed using the guided spectral unmixing feature of the IVIS, a program that utilizes known positive (MS-only solution) and negative (POR-only solution) controls of the desired fluorophore to correct for tissue auto-fluorescence. Identically sized regions of interest (ROIs) were drawn over the entirety of each of the filters, and the average radiant efficiency (RE) within the ROI was measured.

### 2.8. Statistical analysis

Data are presented as means ± standard error of the mean (SEM) as measured by fluorescence (RE) by IVIS or by fluorescent INT by well plate reader. Linear regression was used to evaluate the goodness of fit and trends associated with MS concentrations in filters from both POR- and CON-containing solutions. Analysis of Covariance (ANCOVA) was used to calculate the effects of increasing MS (covariate) on RE or INT and to eliminate these effects to assess differences in means between POR vs. CON, CELL vs. PCTE, and their interaction. All statistical analyses were completed using SAS and GraphPad Prism (version 9.5.1 (733)).

## 3. Results

### 3.1. Characterizing microsphere size and filter surfaces

**Fig. 2.**
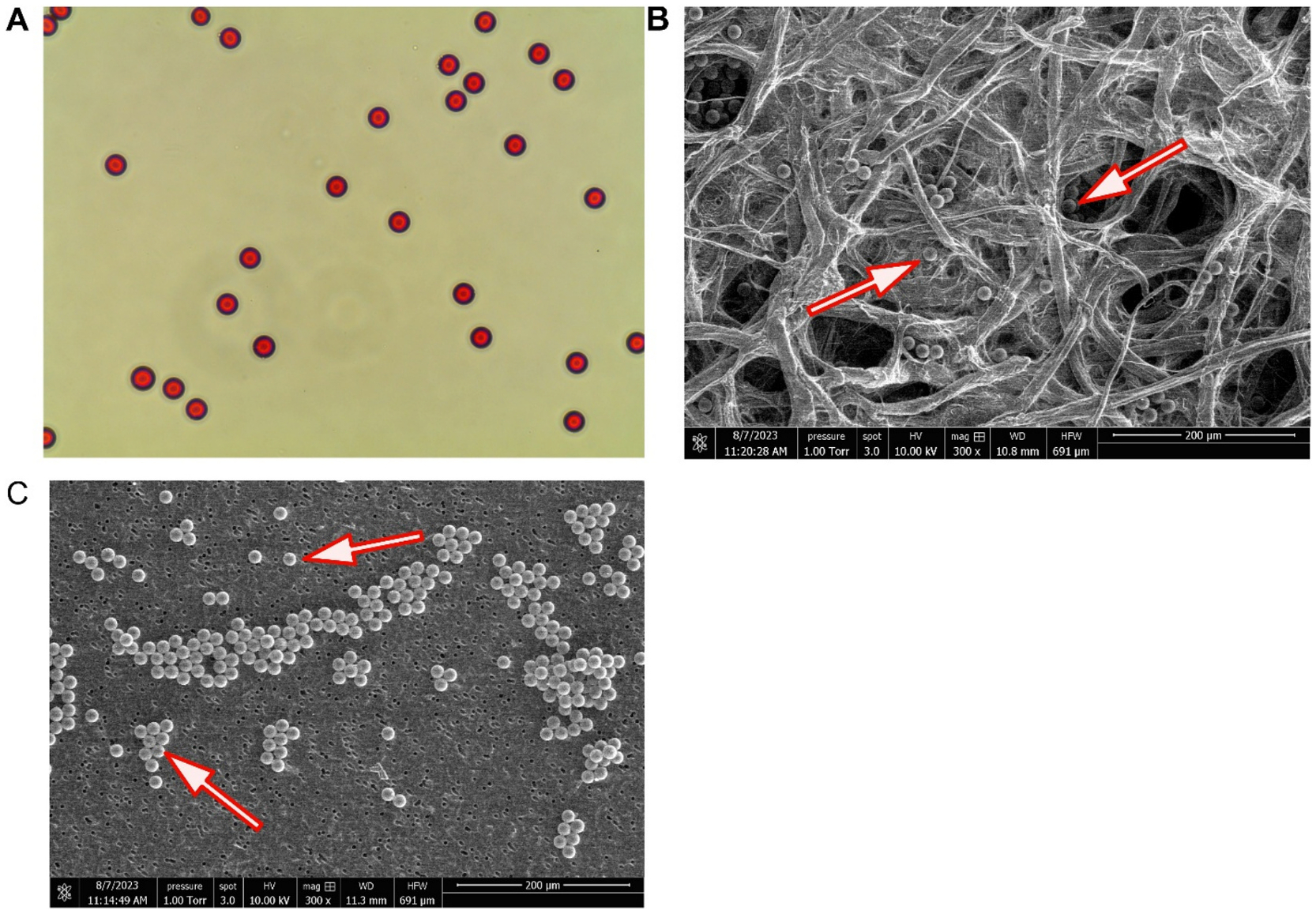
Microscopy of microspheres. a) Light microscopy image of fluorescent MS. b, c) Environmental SEM images (300×) of b) PCTE and c) CELL filters with embedded fluorescent MS (arrows indicate embedded MS). Cellulose = CELL; Microspheres = MS; Polycarbonate = PCTE; Scanning electron microscope = SEM. Created with BioRender.com.

To determine the size and morphological characteristics of the MS, MS were imaged via light microscopy, showing uniform size and spherical shape (Fig. 2A). Low-vacuum ESEM images were taken of the PCTE and CELL filters after having MS filtered through (Fig. 2B and C). The PCTE filter was smooth with 5 μm pores covering its surface, whereas the CELL filter was a coarser surface with fibers layered over each other in random patterns to exclude samples from passing through. The PCTE filter image showed that the MS lay on top of the flat surface of the filter and clustered together in wave-like patterns. In contrast, the CELL filter had a very different filtration interaction with the MS as they were more dispersed and were trapped within the various pockets that formed between the overlaying cellulose fibers. The MS had a diameter of 15.51 ± 0.27 μm as obtained from ESEM images and analyzed using ImageJ (*n* = 20).

### 3.2. Measuring fluorescence in POR and CON samples via standard fluorometric method

To briefly investigate the standard fluorometric method, 1.1 g samples of POR with a set number of MS added were digested, filtered using either a CELL or PCTE filter, incubated with 2-ethoxy ethyl acetate for 7 days, and imaged via well plate reader alongside CON samples. This method is consistent with standard methods for processing tissue samples with embedded MS and results in INT values for each sample [4,6].

**Fig. 3.**
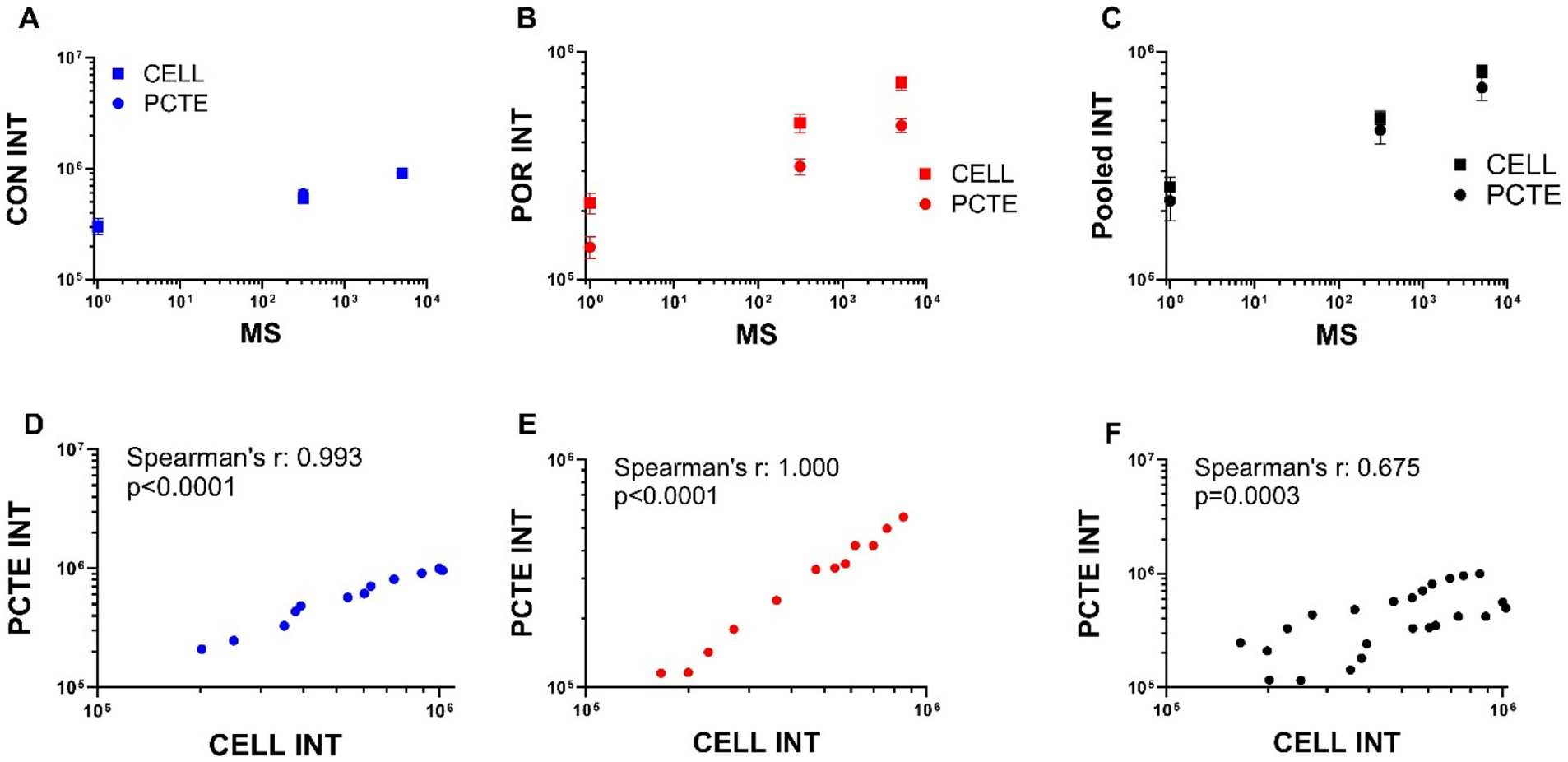
Standard fluorometric measurements of control and porcine samples. Scatter plot representations of fluorometric measurements of a) CON, b) POR, or c) pooled samples filtered through either CELL or PCTE filters. Results are presented as means ± SEM (simple linear regression; *n* = 4). Correlation plots of the fluorescent INT from CELL and PCTE filtered d) CON, e) POR, or f) pooled datasets. Results are presented as raw data (Spearman’s; n = 4). Cellulose = CELL; control = CON; fluorescent intensity = INT; polycarbonate = PCTE; porcine tissue = POR.

The datasets were analyzed by the individual conditions (POR/CELL, POR/PCTE, CON/CELL, and CON/PCTE) and by pooled groups (POR vs. CON and CELL vs. PCTE). Each dataset underwent simple linear regression to determine goodness of fit and to compare trends between conditions. INT between the PCTE and CELL filter conditions was comparable both when using the CON samples and in the pooled dataset (Fig. 3A to C). However, there was a separation in which POR/CELL express higher INT than POR/PCTE across all MS concentrations. Each plot demonstrated strong positive correlations (Spearman’s rho) between CELL and PCTE filters with the CON (*r* = 0.993, *p <* 0.001) and POR samples (*r* = 1.000, p < 0.001) and a lessened positive correlation in the pooled dataset (*r* = 0.675, p < 0.001) (Fig. 3D to F). This reduced correlation is the result of the difference between POR/CELL vs. POR/PCTE values.

**Fig. 4.**
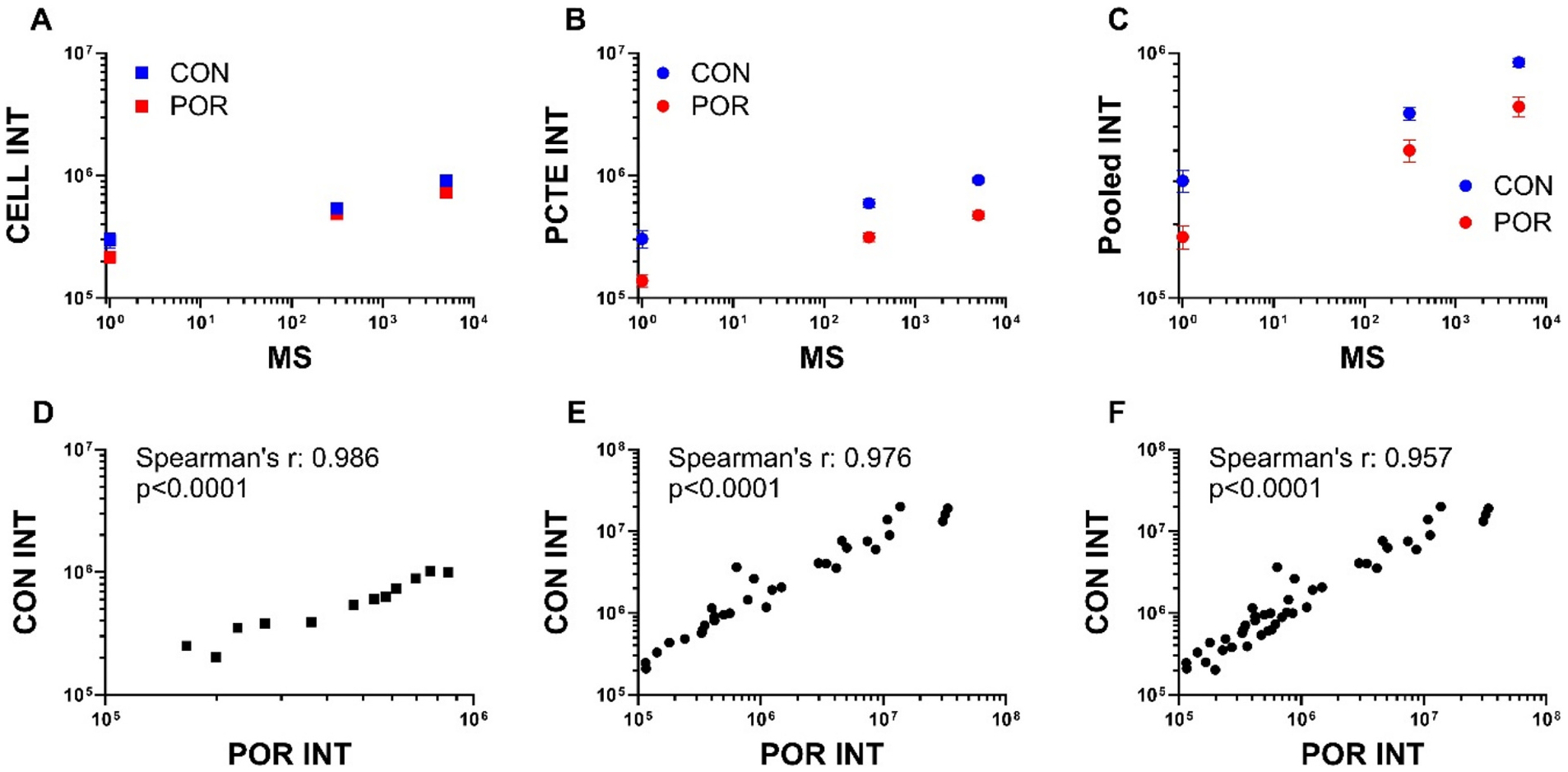
Standard fluorometric measurements of polycarbonate and cellulose filters. Scatter plot representations of fluorometric measurements of a) CELL, b) PCTE, or c) pooled filters with either CON or POR filtered through. Results are presented as means ± SEM (simple linear regression; n = 4). Correlation plots of the fluorescent INT from CON and POR filtered through d) CELL, e) PCTE, or f) pooled filter datasets. Results are presented as raw data (Spearman’s; n = 4). Cellulose = CELL; control = CON; fluorescent intensity = INT; polycarbonate = PCTE; porcine tissue = POR.

Comparisons performed between the POR and CON conditions in either PCTE or CELL filters show that INT in POR samples was lower than CON samples (Fig. 4A to C). This, alongside the noted discrepancy between CELL and PCTE filters when filtering POR samples in Fig. 3B, suggests an interaction between POR samples and PCTE filters as POR/PCTE samples appeared to be dampened in all comparisons. This interaction may be explained by the observation that the PCTE filters degraded in the 2-ethoxy ethyl acetate solution. The likely result of this degradation is that some residual, unfiltered POR protein as well as PCTE particulate was released, retained in the sample placed in the well plate, and dampened the resulting INT. Correlation analyses found strong positive correlations within the CELL filter (*r* = 0.986, *p <* 0.001), PCTE filter (*r* = 0.976, p < 0.001), and the pooled filter data (*r* = 0.957, *p <* 0.001) (Fig. 4D to F).

ANCOVA was used to compare the results of each condition (PCTE vs. CELL, POR vs. CON, and their interaction effects), independent of MS concentration. As expected, increasing MS concentration was found to have a significant linear effect on increasing INT in all conditions (p < 0.001). Adjusted results showed that, while the difference between CELL and PCTE filters was not significant, a trend toward increased INT when using CELL filters was observed (*p* = 0.065). When comparing POR and CON samples, results were significantly different (p < 0.001), with POR samples fluorescing lower than CON samples. There was a significant interaction effect between filter types and samples, with CON/CELL and POR/CELL filters having similar fluorescence and POR/PCTE filters having lower fluorescence compared with CON/PCTE across the MS range (*p* = 0.016). This provides further evidence that the degrading PCTE filters release POR protein into the measured aliquot of each sample and dampen the INT of the POR/PCTE condition. These results show that, while the standard fluorometric method was somewhat insensitive to the filter type, this method of measuring tissue samples has dampening effects, likely due to residual POR tissue on the filter that is released as it degrades and as well as PCTE particulate from the degraded filter, thus raising concerns with use for tissue samples.

### 3.3. Sensitivity analysis of direct imaging method

**Fig. 5.**
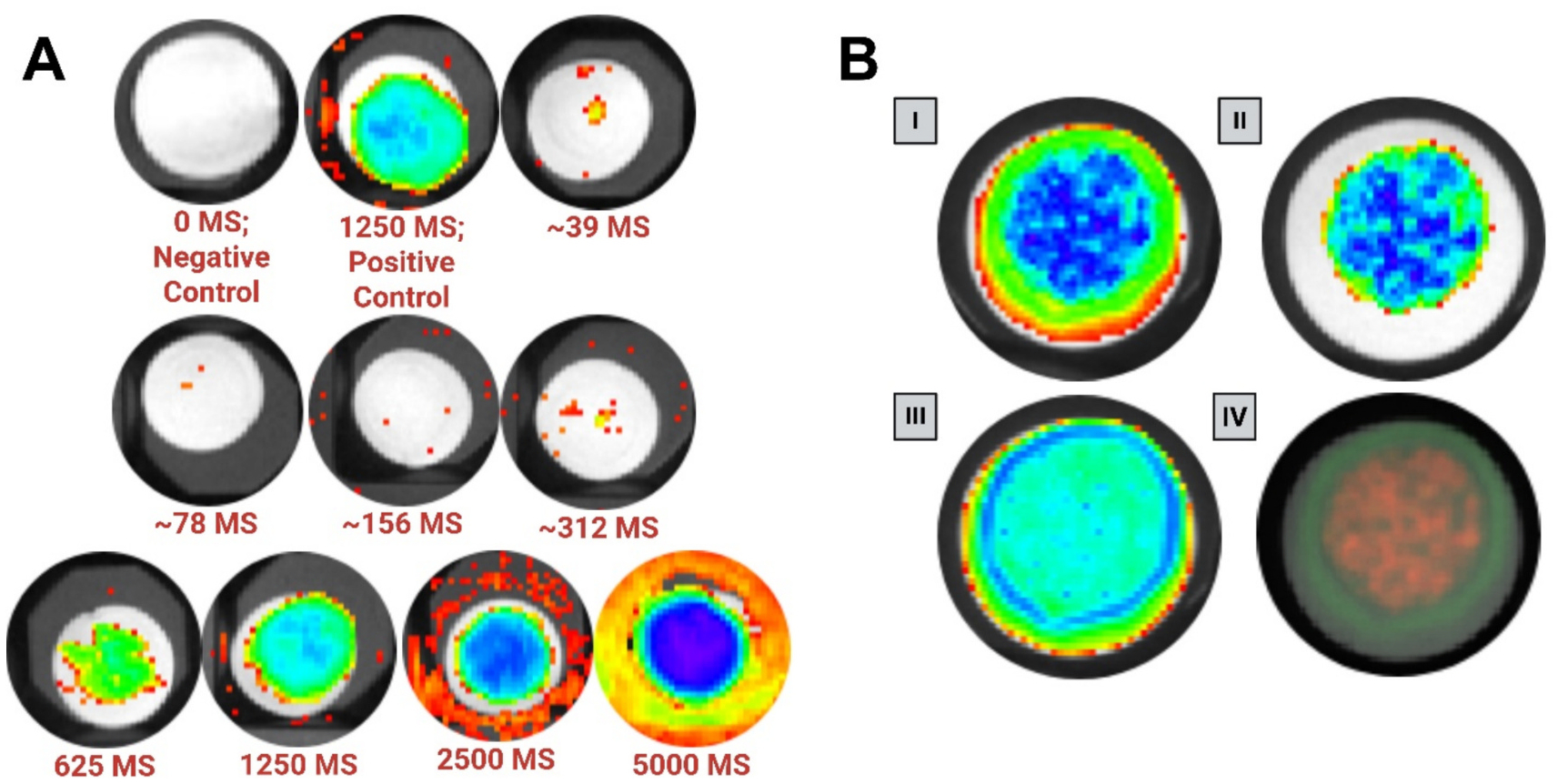
Representative images from the direct imaging method. a) IVIS representative image of POR/CELL samples. b) Spectrally unmixed representative images of i) raw fluorescence, ii) unmixed MS, iii) unmixed autofluorescence, and iv) overlay of autofluorescence (green) and MS (red). Created with BioRender.com.

**Fig. 6.**
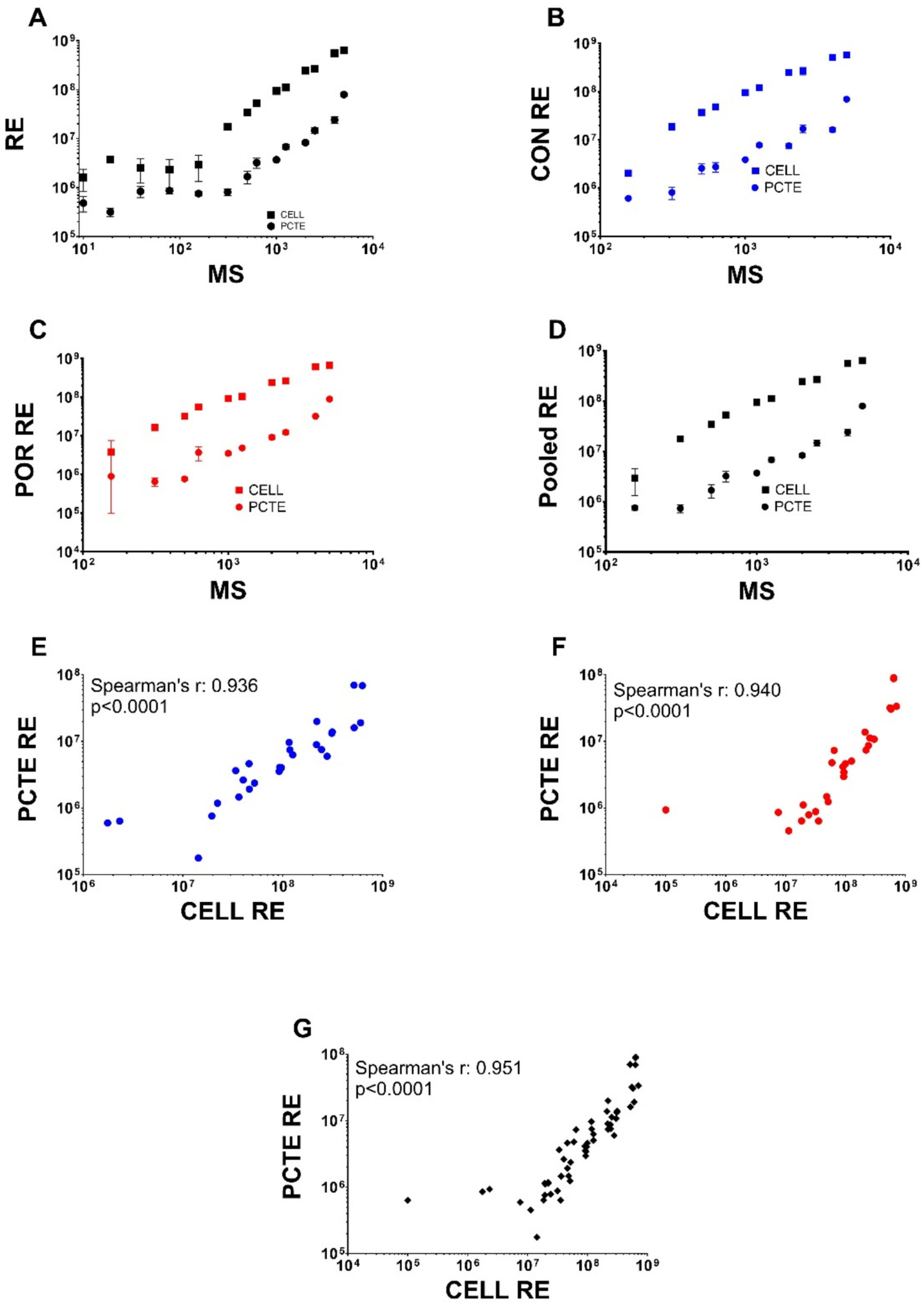
Direct imaging method sensitivity and filter comparison. a) Sensitivity analysis of all filtered samples, with linear fits from the ranges of 10–156 MS and 156–5000 MS in both CELL and PCTE filter conditions. Scatter plot representations of direct imaging measurements of b) CON, c) POR, or d) pooled samples filtered through either CELL or PCTE filters. Results are presented as means ± SEM (simple linear regression; *n* = 3). Correlation plots of the fluorescent INT from CELL and PCTE filtered e) CON, f) POR, or g) pooled datasets. Results are presented as raw data (Spearman’s; n = 3). Cellulose = CELL; control = CON; microspheres = MS; polycarbonate = PCTE; porcine tissue = POR; radiant efficiency = RE.

POR and CON samples with set MS amounts were filtered using CELL or PCTE filters and then imaged using IVIS alongside positive (MS-only) and negative (digested POR-only) controls (Fig. 5A). IVIS images of PCTE or CELL filters were acquired for each concentration of MS, spectrally unmixed using the positive and negative controls, and RE within each ROI was recorded and compared between CELL and PCTE filters. A sensitivity analysis was then performed on the full span of MS (0–5000) (Fig. 6A).

MS values were broken into two ranges: the ‘insensitive’ range of 0–156 MS where the null hypothesis (slope = 0) could not be rejected (PCTE R^2^ = 0.310, *p* = 0.329; CELL R^2^ = 0.025, *p* = 0.799) and the ‘sensitive’ range where a linear relationship between RE and MS numbers could be made (PCTE R^2^ = 0.7726, *p <* 0.001; CELL R^2^ = 0.9901, p < 0.001). Notably, PCTE had a poorer R^2^ value as well as a greater SEM, indicating greater variance among samples in comparison with CELL samples. This sensitivity analysis determined that the direct imaging method of measuring embedded MS samples described in this paper was sensitive for samples with a minimum of 156 MS. Thus, the results described below are of samples with this minimum (156–5000 MS).

### 3.4. Measuring fluorescence directly with CELL or PCTE filters

To test our hypothesis that directly imaging samples containing intact MS after filtration through CELL filters will yield equivalent sensitivity in comparison with the conventional PCTE filter material, we made comparisons between sample types (Fig. 6B to D). Both filters retained good fits with each sample type (POR/CELL R^2^ = 0.966; POR/PCTE R^2^ = 0.777; CON/CELL R^2^ = 0.930; CON/PCTE R^2^ = 0.690), but the PCTE filters had notably poorer fits, indicating a greater variance among samples. In addition, CELL filters had significantly higher RE than PCTE filters at each concentration of MS. Some divergence from the linear regression was noted between the 156 and 312 MS-containing samples, suggesting that the actual minimum sensitivity of this method is likely in that range.

Each correlation plot demonstrated strong positive correlations between the CELL and PCTE filters in the CON (*r* = 0.936, *p <* 0.001), POR (*r* = 0.940, p < 0.001), and pooled datasets (*r* = 0.951, p < 0.001), with the correlations having the slopes of 0.073, 0.089, and 0.082 respectively (Fig. 6E to G). These low slope values show that CELL samples have greater RE at each point than PCTE samples (i.e., while both filters fluoresce more with increased MS amounts, the CELL filters fluoresce more strongly). ANCOVA confirmed that the difference between CELL and PCTE filters was significantly different (p < 0.001). These results show that either the CELL filters amplify or the PCTE filters dampen the RE signal from the filtered MS, regardless of sample type or MS concentration.

### 3.5. Measuring fluorescence in POR and CON filters directly

**Fig. 7.**
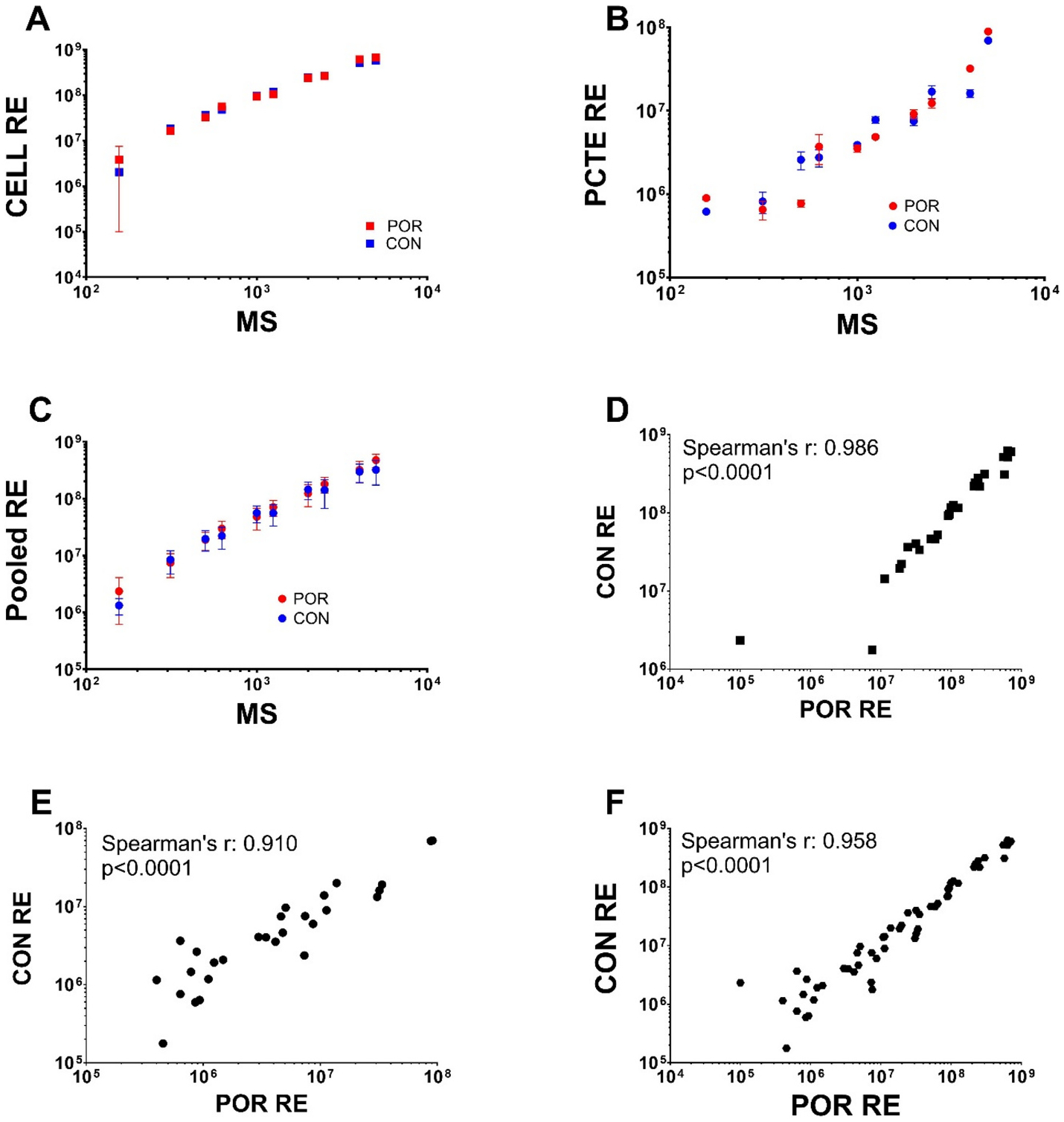
Direct imaging method porcine and control comparison. Scatter plot representations of direct imaging measurements of a) CELL, b) PCTE, or c) pooled filters with either CON or POR filtered through. Results are presented as means ± SEM (simple linear regression; n = 3). Correlation plots of the fluorescent INT from CON and POR filtered through d) CELL, e) PCTE, or f) pooled filter datasets. Results are presented as raw data (Spearman’s; n = 3). Cellulose = CELL; control = CON; microspheres = MS; polycarbonate = PCTE; porcine tissue = POR; radiant efficiency = RE.

Spectral unmixing, an algorithmic tool provided by the Living Image software suite (PerkinElmer, Inc., MA), can remove autofluorescence and allow for separation of various fluorescent signals in a single sample. We wanted to determine if this could allow direct imaging of POR samples as POR may auto-fluoresce significantly in comparison to CON samples. This autofluorescence is due to the wide range of dissolved proteins and biological products present in POR, many of which fluoresce at specific spectra close to the desired spectra for the MS. Thus, POR and CON samples were imaged, spectrally unmixed, and data were compared (Fig. 5B). The comparison of sample type, sorted by filter, showed that the linear fits (POR/CELL R^2^ = 0.966; CON/CELL R^2^ = 0.930; POR/PCTE R^2^ = 0.777; CON/PCTE R^2^ = 0.690) appeared to be equivalent between POR and CON samples in CELL filters and in the pooled dataset with divergence around 312 MS, while the PCTE filter showed equivalence between POR and CON samples only around 1000 MS and divergence at +/− 1000 MS (Fig. 7A to 7C). These results provide further evidence that PCTE filters introduce greater variance to samples that are imaged directly and that the sensitivity of the direct imaging method to estimate MS quantity using CELL filters is between 156 and 312 MS.

Each correlation plot demonstrated strong positive correlations between the POR and CON samples in the CELL (*r* = 0.986, *p <* 0.001), PCTE (*r* = 0.910, p < 0.001), and pooled (*r* = 0.959, p < 0.001) datasets, with slopes of 0.829, 0.730, and 0.9576 respectively (Fig. 7D to G). These slope values indicate that the relationship between POR and CON samples is nearly 1:1 in RE as samples increase in MS concentration (i.e., as the MS increases, both filters’ RE increase at an equivalent rate). ANCOVA found that the difference between the POR and CON samples was not significantly different (*p* = 0.651). In addition, no interaction effect between sample type and filter type was found, unlike in the standard fluorometric method. These results show that spectrally unmixing samples eliminates unwanted auto-fluorescence and allows for accurate measurement of the RE of each sample’s MS quantity.

### 3.6. Correlation between standard fluorometric and direct imaging measurements

**Fig. 8.**
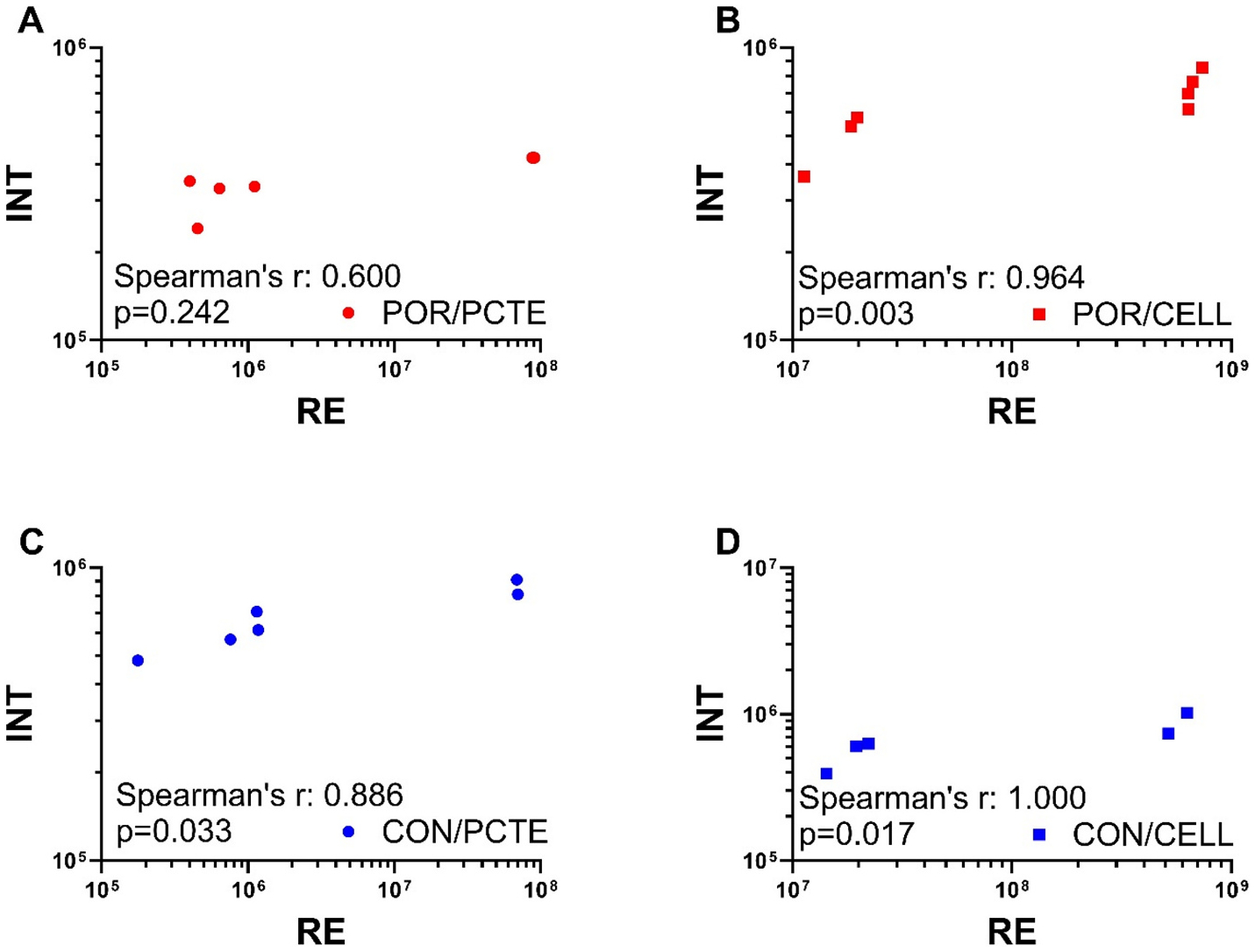
Correlations between standard fluorometric and direct imaging methods. a-d) Correlation graphs depicting the correlative relationship between the RE of the direct imaging method with the INT of the standard fluorometric method in POR/PCTE, POR/CELL, CON/PCTE, and CON/CELL. Results are presented as raw data (Spearman’s; n = 4). Cellulose = CELL; control = CON; fluorescent intensity = INT; polycarbonate = PCTE; porcine tissue = POR; radiant efficiency = RE.

Samples from both the standard fluorometric and the direct imaging methods were plotted against each other to determine correlation between the methods (Fig. 8A to 8D). The PCTE filtered samples continued to show poorer results with weaker correlations and significantly higher *p*-values (POR/PCTE, *r* = 0.600, *p* = 0.242; CON/PCTE, *r* = 0.886, *p* = 0.033) compared to the CELL filtered samples (POR/CELL, *r* = 0.964, *p* = 0.003; CON/CELL, *r* = 1.000, *p* = 0.017). The POR/PCTE condition failed to find any significant correlation between the standard fluorometric and the direct imaging measurements.

### 3.7. Cost analysis

**Fig. 9.**
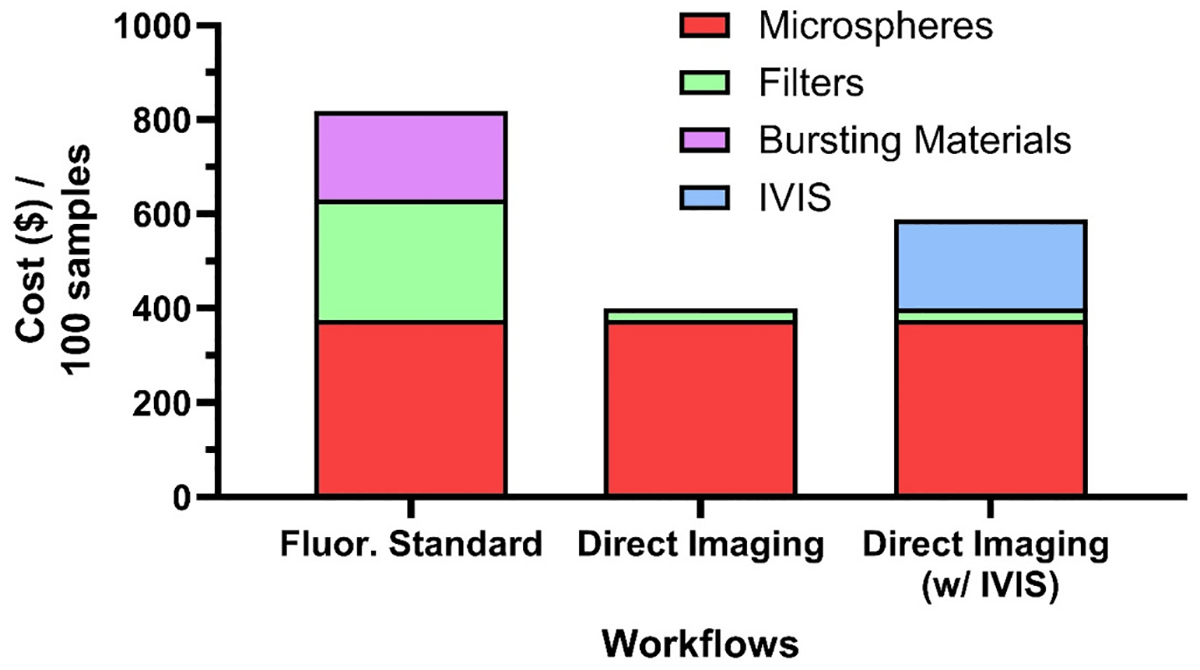
Cost comparison. Analysis of cost for the standard fluorometric, direct imaging, and direct imaging with the costs of IVIS imaging methods per 100 samples.

A further comparison between the two protocols was made with respect to the cost spent for the full imaging pipeline, from sample preparation to measurement, with the IVIS imaging separated as some lab spaces may not require fees to image with such equipment (Fig. 9). The shared cost between both methods was the fluorescent MS themselves ($375/100 samples). Notably, the direct imaging method filter costs (using CELL filters) were found to be significantly lower than those used in the standard fluorometric method (using PCTE filters) ($24.50 vs. $256/100 samples). Furthermore, the standard fluorometric method required the ordering of polypropylene tubes, serological pipettes, 96-well plates, and 2-EEA to burst the MS significantly increasing the cost as well ($186.85/100 samples). The direct imaging method also had its own unique costs with the usage of the IVIS system ($188/100 samples). Overall, the direct imaging method was found to be the least expensive method ($399.5/100 samples and $587.5/100 samples with the cost of IVIS) with a 51.1 % and 28.2 % decrease in cost respectively compared to the standard fluorometric method ($817.85/100 samples), providing a significant improvement.

## 4. Discussion

The standard method for fluorescent perfusion imaging consists of filtering the fluorescent MS of each tissue sample through a PCTE filter, chemically bursting the MS to release the fluorescent dye, and fluorometrically measuring the fluorescence of each sample. This is an expensive process that carries the potential for MS loss due to its complexity and requires various specialized equipment. We developed a simpler method for directly imaging tissue samples using a CELL filter with direct IVIS imaging, expecting that it would reduce the necessary expenses involved as well as retain sensitivity to reported literature values. Critically, we found that the CELL filter performed better than the conventional PCTE filter for direct imaging, providing a better fit and improved qualitative properties.

### 4.1. Comparison between CELL and PCTE filter properties

PCTE filters were shown to have a greater variance when directly imaging the filters and had a poorer fit for both sample types (Table 1). In addition to this poor performance, it was found that the PCTE filters had a significantly lower RE signal in comparison to the CELL filter samples. The correlation plots for the POR, CON, and pooled datasets indicated an 11- to 13-fold difference between CELL and PCTE filters across the MS gradient. The most likely factor for this difference is the physical and optical characteristics of the filters themselves. The PCTE filter is a smooth, semi-translucent, thin, white filter, whereas the CELL filter is a rough, opaque, thick, white filter. We suspect that the PCTE filter’s semi-translucency allowed emitted photons from the fluorescent MS to pass through the filter and strike the black weigh boats, dampening the resulting RE [18]. In contrast, the CELL filter’s opacity prevented photons from passing through and instead reflected them back toward the detectors, effectively amplifying the RE, which in turn increased the signal-to-noise ratio, which also partially explains the lowered variance in the CELL dataset [19,20].

With respect to the variance and fit discrepancies, we theorize that it may also be partially due to poorer capture of the MS between the filter types, as the MS became more effectively trapped between the fibers of the CELL filters, leading to better adherence to the rough surface. In contrast, the MS on the PCTE filters were loosely attached to the filter surface and may have adsorbed to the glass filtration cylinder as the setup was disassembled or physically fallen off of the filter due to a lack of strong adhesion to the filter surface, leading to less consistent measurements. This is conjecture, albeit informed by the results and established optical theory, but we believe that effective trapping and filter opacity account for the increased RE measurements from CELL filters as well as the increased variance among PCTE filters. Further qualitative differences between the filters were observed in both the standard fluorometric method and the direct imaging method. Due to their thin and lightweight characteristics, the PCTE filters were fragile and prone to flying off surfaces with minute airflow. In comparison, the CELL filters were thicker and heavier than the PCTE filters, allowing for simple removal from the base, easy storage, and transportation.

### 4.2. Unmixing tissue autofluorescence from fluorescent signal

Tissue auto-fluorescence is always an issue when analyzing fluorescent signals with tissue particulate present, as it significantly adds to the background fluorescence of an image and must be either accounted for or removed. This represented a major hurdle in developing the direct imaging method, so a two-pronged solution was developed: (1) use a MS with excitation/emission spectra beyond the most significant auto-fluorescent ranges (Ex/Em: 200–450/300–550); and (2) utilize the spectral unmixing feature of the Living Image software suite to remove tissue autofluorescence [21]. Despite choosing a MS that had minimal overlap, tissue auto-fluorescence was visibly present and needed to be eliminated via spectral unmixing. This was likely due to imperfect digestion, in which protein aggregates larger than the pore size of the filters remained or reformed after the digestion. It also may have been due to fat globules, which, despite surfactant treatment, likely formed micelles larger than the pore size of the filter and adhered to the surface.

The results show that spectral unmixing was a critical process that eliminated the effects of auto-fluorescence, allowing for accurate measurement of MS on filters. The statistical analysis showed that the POR and CON samples were not significantly different, except for when they were filtered with the PCTE filters, which have been shown to be less reliable than the CELL filters for the direct imaging method. In addition to not being significantly different, the correlation plots of the CELL and pooled datasets showed a near 1:1 relationship between POR and CON sample RE. This demonstrates the efficacy of spectral unmixing for removing tissue auto-fluorescence and ensures accurate measurement of MS quantities in both POR and CON samples.

### 4.3. Comparing trends in the standard fluorometric method with direct imaging

The resource-intensive standard fluorometric method was tested and used for comparison with the direct imaging method proposed in this study. To that end, a limited dataset of 0, 312, and 5000 MS were measured in all filter/sample combinations to determine trends. The most critical trend was that of the POR samples, which had a dampened signal in both filter types, although the PCTE filter had a more pronounced dampening effect. It was found that the sample type had a significant effect on the measured INT across the MS gradient, as did the interaction between the filter and sample type. This, along with the dampened POR signal, indicated that the PCTE filter likely retained some tissue particulate or proteins from the POR samples, which were released when the filter degraded, and the tissue and filter particulate dampened the fluorescent signal when being measured by the fluorometer. This effect was likely less apparent in the CELL filter samples as the CELL filters were comprised of a thick mesh of fibers that may more readily adsorb the unfiltered POR protein and keep them trapped as the MS are chemically burst. This sensitivity to sample type indicates that the standard fluorometric method may be a poor method for measuring perfusion in tissue samples as the interaction between tissue and measured signal likely will result in error of MS quantification.

In comparison, the direct imaging method was determined to be insensitive to sample type, was found to lack an interaction between filter type and sample type that affected the measured signal, and that, while filter type affects RE measurement, it likely serves as an amplifying effect as CELL filters show good fit and minimal variance in comparison to the PCTE filters. The correlation between the direct imaging method and the standard fluorometric method further provided evidence that PCTE filters had poorer performance in comparison to CELL filters as the CON/PCTE condition had a poorer correlation than either CELL condition and the POR/PCTE condition lacked a significant correlation between the two imaging methods.

### 4.4. Examining the ‘rule of thumb’ MS sensitivity

The standard sensitivity of perfusion studies is typically cited as ~400 MS per sample of the organ or tissue of interest [12,15]. Interestingly, this sensitivity is not based on the detection limit, which was determined first with radioactive MS and has since been used as the ‘rule of thumb’ for all MS used in perfusion imaging. This value was determined from how the number of MS in an organ or tissue sample can be modeled by the Poisson distribution, and 400 MS is the minimum necessary to have 95 % confidence that the measured perfusion rate is within 10 % of the true perfusion rate. However, this ‘rule of thumb’ for MS perfusion imaging is not exactly accurate, as determined by Polissar et al. (2000) [22]. This group found that mathematical adjustments for noise heterogeneity could significantly reduce the limit and that a better ‘rule of thumb’ is that a total of 15,000 MS in the entire organ or tissue of interest or approximately 200 MS/sample are necessary for accurate estimates of heterogeneity. While this work has not been replicated with fluorescent MS, fluorescent MS have been shown to have strong linearity with radioactive MS and so the sensitivity is likely lower than the ‘rule of thumb’ used as well [12].

In comparison, our direct imaging method has been found to be sensitive up to the range between 156 and 312 fluorescent MS and shows a strong linear relationship between increasing MS quantities and the fluorescent signal measured. In addition, this method allows for better fidelity in measuring low MS values due to the amplification effects of the CELL filter discussed above. This sensitivity is notably lower than the 400 MS/sample ‘rule of thumb’ used in MS perfusion imaging while still being within the 200 MS/sample limit Polissar et al. determined and thus should be applicable to the current field of perfusion imaging.

### 4.5. Qualitative benefits of direct imaging

The novel direct imaging method described in this work significantly reduced costs when compared to the standard fluorometric method. Further qualitative benefits of the direct imaging method are the reduced complexity of the processing as well as the removal of the need for 2-ethoxy ethyl acetate. Lowered complexity translates to a lowered potential for human error and a faster training time for new users of the method, both critical for the processing of large quantities of samples necessary for regional perfusion studies. The removal of 2-ethoxy ethyl acetate is a critical benefit as it dissolves polystyrene, a common plastic used in lab work, and can soften other plastics. In addition to its effects on plastics, it is also an irritant and a teratogen [23]. Due to these characteristics, 2-ethoxy ethyl acetate needs to be handled underneath a fume hood with non-reactive materials such as polypropylene or glass pipettes and vials and polypropylene well plates. By removing this chemical from the protocol, the direct imaging method removes a workplace hazard as well as allowing the use of standard labware made of synthetic polymers.

### 4.6. Study limitations

The work described here is preclinical and was designed to investigate and describe a potential new method of detecting fluorescent microspheres for perfusion imaging through direct imaging of the filtered samples. The data presented here provides the necessary information to provide an understanding of the weaknesses and strengths of the standard and the new imaging methods as well as detail how to utilize the direct imaging method for future work in perfusion imaging of various in vivo models. This study provided only a ex vivo model of large animal regional organ perfusion using a single-color MS and so future works should involve true in vivo validation as well as the use of various colors of MS to determine which fluorescent signals are optimal for this method. It should be noted that, while this study provided conjectures with regards to variance discrepancies, optical characteristics of the filters, and the minimum MS necessary for a sample, these were based on the data gathered and a full investigation of these conjectures are beyond the scope of this work and may provide avenues for future work.

### 4.7. Conclusions

In summary, this direct imaging method for measuring perfusion with fluorescent MS has significant advantages over the conventional fluorometric method. Specifically, the direct imaging method was found to be insensitive to the presence of undigested tissue which may not filter through and interfere with the standard fluorometric method. The direct imaging method was also found to have removed a significant interaction between tissue and the standard plastic (PCTE) filter that affected measured fluorescent intensity that was noted in the standard fluorometric method. In addition to proposing a novel measurement method, the present study found that simple cellulose (CELL) filters are a significant improvement over the standard and more costly PCTE filters. CELL filters retained a better linear fit along a gradient of MS concentrations, amplified the measured fluorescent signal, and demonstrated significantly better qualitative characteristics.

Overall, it was found that the use of the direct imaging method with CELL filters provided improved sensitivity to the currently accepted ‘minimum’ of 400 MS per tissue sample with minimal interference from filter debris, unfiltered protein aggregates, and possible MS loss. This, coupled with the significantly lowered cost, provides strong evidence that the direct imaging method will provide an improvement over the industry standard for fluorescent perfusion measurement.

## Figures and Tables

**Table 1:** Comparison between CELL and PCTE filter properties.

	Physical Characteristics	Surface Texture	Signal Properties	Variance and Fit
PCTE	Semi-translucent, thin, fragile	Smooth, porous	Lower signal	Poor fit, high variance
CELL	Opaque, thick, sturdy	Rough, fibrous	Higher Signal (11–13×)	Improved fit, lower variance

## Data Availability

Data will be made available on request.
